# Quadrupling Muscle Mass in Mice by Targeting TGF-ß Signaling Pathways

**DOI:** 10.1371/journal.pone.0000789

**Published:** 2007-08-29

**Authors:** Se-Jin Lee

**Affiliations:** Department of Molecular Biology and Genetics, Johns Hopkins University School of Medicine, Baltimore, Maryland, United States of America; Katholieke Universiteit Leuven, Belgium

## Abstract

Myostatin is a transforming growth factor-ß family member that normally acts to limit skeletal muscle growth. Mice genetically engineered to lack myostatin activity have about twice the amount of muscle mass throughout the body, and similar effects are seen in cattle, sheep, dogs, and a human with naturally occurring loss-of-function mutations in the myostatin gene. Hence, there is considerable interest in developing agents capable of inhibiting myostatin activity for both agricultural and human therapeutic applications. We previously showed that the myostatin binding protein, follistatin, can induce dramatic increases in muscle mass when overexpressed as a transgene in mice. In order to determine whether this effect of follistatin results solely from inhibition of myostatin activity, I analyzed the effect of this transgene in myostatin-null mice. *Mstn^−/−^* mice carrying a follistatin transgene had about four times the muscle mass of wild type mice, demonstrating the existence of other regulators of muscle mass with similar activity to myostatin. The greatest effect on muscle mass was observed in offspring of mothers homozygous for the *Mstn* mutation, raising the possibility that either myostatin itself or a downstream regulator may normally be transferred from the maternal to fetal circulations. These findings demonstrate that the capacity for increasing muscle growth by manipulating TGF-ß signaling pathways is much more extensive than previously appreciated and suggest that muscle mass may be controlled at least in part by a systemic mode of action of myostatin.

## Introduction

Myostatin (MSTN) is a transforming growth factor-ß (TGF-ß) family member that plays a critical role in regulating skeletal muscle mass [1]. Mice engineered to carry a deletion of the *Mstn* gene have about a doubling of skeletal muscle mass throughout the body as a result of a combination of muscle fiber hyperplasia and hypertrophy [2]. Moreover, loss of myostatin activity resulting either from postnatal inactivation of the *Mstn* gene [3], [4] or following administration of various myostatin inhibitors to wild type adult mice [5]–[7] can also lead to significant muscle growth. Hence, myostatin appears to play as least two distinct roles, one to regulate the number of muscle fibers that are formed during development and a second to regulate growth of muscle fibers postnatally. The function of myostatin appears to have been conserved across species, as inactivating mutations in the myostatin gene have been demonstrated to cause increased muscling in cattle [8]–[11] , sheep [12], dogs [13] and humans [14]. As a result, there has been considerable effort directed at developing strategies to modulate myostatin activity in clinical settings where enhancing muscle growth may be beneficial. In this regard, loss of myostatin activity has been demonstrated to improve muscle mass and function in dystrophic mice [15]–[17] and to have beneficial effects on fat and glucose metabolism in mouse models of obesity and type II diabetes [18].

Myostatin is synthesized as a precursor protein that undergoes proteolytic processing to generate an N-terminal propeptide and a C-terminal dimer, which is the biologically active species. Following proteolytic processing, the propeptide remains bound to the C-terminal dimer and maintains it in an inactive, latent complex [6], [19], [20], which represents one of the major forms of myostatin that circulates in the blood [21], [22]. In addition to the propeptide, other binding proteins are capable of regulating myostatin activity *in vitro*, including follistatin [19], [21], FLRG [22], and Gasp-1 [23]. We previously showed that follistatin can also block myostatin activity *in vivo*; specifically, we showed that follistatin can ameliorate the cachexia induced by high level expression of myostatin in nude mice [21] and that transgenic mice expressing follistatin in muscle have dramatic increases in muscle mass [19]. Here, I show that overexpression of follistatin can also cause substantial muscle growth in mice lacking myostatin, demonstrating that other TGF-ß related ligands normally cooperate with myostatin to suppress muscle growth and that the capacity for enhancing muscle growth by targeting this signaling pathway is much larger than previously appreciated.

## Results

### Increased muscle mass in transgenic mice expressing FLRG

**Figure 1:**
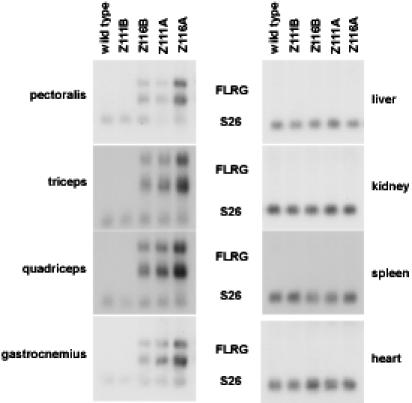
Northern analysis of FLRG transgenic mice. Total RNA was prepared from various tissues from 10-week old female mice, electrophoresed, blotted, and probed with a fragment derived from SV40 corresponding to the processing/polyadenylation sequences present in the transgenic construct. The blots were re-hybridized with a probe for the S26 ribosomal protein to control for loading.

Previous studies have identified several proteins that are normally found in a complex with myostatin in the blood [22], [23]. One of these is the follistatin related protein, FLRG, which has been demonstrated to be capable of inhibiting myostatin activity *in vitro*. To determine whether FLRG can also inhibit myostatin activity *in vivo*, I generated a construct in which the FLRG coding sequence was placed downstream of a myosin light chain promoter/enhancer. From pronuclear injections of this construct, a total of four transgenic mouse lines (Z111A, Z111B, Z116A, and Z116B) were obtained containing independently segregating insertion sites. Each of these four transgenic lines was backcrossed at least 6 times to C57 BL/6 mice prior to analysis in order to control for genetic background effects. Northern analysis revealed that in three of these lines the transgene was expressed in skeletal muscles but not in any of the non-skeletal muscle tissues examined (Figure 1); in the fourth line, Z111B, the expression of the transgene was below the level of detection in these blots. As shown in Table 1, all four lines exhibited significant increases in muscle weights compared to wild type control mice. These increases were observed in all four muscles that were examined as well as in both sexes. Moreover, the rank order of magnitude of these increases correlated with the rank order of expression levels of the transgene; in the highest-expressing line, Z116A, muscle weights were increased by 57–81% in females and 87–116% in males compared to wild type mice. Hence, FLRG is capable of increasing muscle growth in a dose-dependent manner when expressed as a transgene in skeletal muscle.

**Table 1:** Muscle weights (mg) of FLRG (Z) and follistatin (F66) transgenic mice.

	offspring	sex	mother	n	pectoralis	triceps	quadriceps	gastrocnemius
1	*Mstn* ***^+/+^***	F	*Mstn^+/+^*	22	47.3±0.8	68.2±1.1	142.8±1.7	95.9±1.3
2	*Mstn* ***^+/−^***	F	*Mstn^+/−^*	15	63.0±0.8 a	90.0±1.5 a	176.6±2.4 a	122.8±1.6 a
3	*Mstn* ***^−/−^***	F	*Mstn^+/−^*	10	105.7±3.4 a	148.7±3.7 a	266.9±6.7 a	181.1±3.8 a
4	Z111B	F	N/A	11	53.5±1.5 b	75.3±2.2 b	151.2±4.2	102.7±2.8 c
5	Z116B	F	N/A	15	64.9±1.2 a	98.3±2.0 a	200.2±3.8 a	141.5±3.1 a
6	Z111A	F	N/A	12	69.8±3.1 a	105.4±4.6 a	223.9±8.3 a	160.6±6.7 a
7	Z116A	F	N/A	11	74.4±2.1 a	116.6±3.8 a	236.5±5.8 a	173.6±5.0 a
8	Z116A, *Mstn* ***^+/−^***	F	*Mstn^+/−^*	8	93.0±1.8 a	151.4±4.4 a	295.4±7.3 a, d	224.9±6.6 a, e
9	*Mstn* ***^+/+^***	M	*Mstn^+/+^*	19	73.5±1.3	91.5±1.6	190.0±3.2	129.4±1.7
10	*Mstn* ***^+/−^***	M	*Mstn^+/−^*	13	94.3±2.0 f	127.1±2.6 f	243.2±5.4 f	167.5±3.2 f
11	*Mstn* ***^−/−^***	M	*Mstn^+/−^*	10	190.8±7.1 f	236.1±5.2 f	390.1±9.4 f	272.6±4.9 f
12	Z111B	M	N/A	10	78.5±1.8 g	99.4±2.2 h	199.9±3.9	135.0±2.9
13	Z116B	M	N/A	11	98.6±3.9 f	131.1±4.3 f	267.1±8.5 f	188.8±5.3 f
14	Z111A	M	N/A	9	113.7±6.4 f	156.4±9.5 f	307.4±15.5 f	221.3±10.4 f
15	Z116A	M	N/A	11	137.3±6.7 f	196.5±5.9 f	370.5±14.0 f	279.5±10.4 f
16	*F66, Mstn* ***^+/+^***	M	*Mstn^+/+^*	20	121.9±2.3 f	182.6±5.0 f	440.6±11.1 f	295.3±5.6 f
17	*F66, Mstn* ***^+/+^***	M	*Mstn^+/−^*	23	126.5±2.6	186.6±4.5	480.7±11.6 i	314.7±6.7 i
18	*F66, Mstn* ***^+/−^***	M	*Mstn^+/−^*	12	185.4±6.1 j	307.2±8.9 j	583.7±19.2 j	384.3±10.9 j
19	*F66, Mstn* ***^+/−^***	M	*Mstn^−/−^*	11	200.3±5.9	306.5±9.6	637.4±12.5 k	439.3±9.8 l
20	*F66, Mstn* ***^−/−^***	M	*Mstn^+/−^*	14	280.1±7.7 l	383.7±9.2 l	619.7±16.0 l	492.1±13.4 l
21	*F66, Mstn* ***^−/−^***	M	*Mstn^−/−^*	15	320.1±9.0 m,o	412.1±4.6 m,o	668.9±8.2 n,o	529.6±10.1 n,o

a *p*<0.001 vs. line 1,

b *p*<0.01 vs. line 1,

c *p*<0.05 vs. line 1,

d *p*<0.01 vs. line 3,

e *p*<0.001 vs. line 3,

f *p*<0.001 vs. line 9,

g *p*<0.05 vs. line 9,

h *p*<0.01 vs. line 9,

i *p*<0.05 vs. line 16,

j *p*<0.001 vs. line 17,

k *p*<0.05 vs. line 18,

l *p*<0.001 vs. line 18,

m *p*<0.01 vs. line 20,

n *p*<0.05 vs. line 20,

o *p*<0.001 vs. line 11

**Figure 2:**
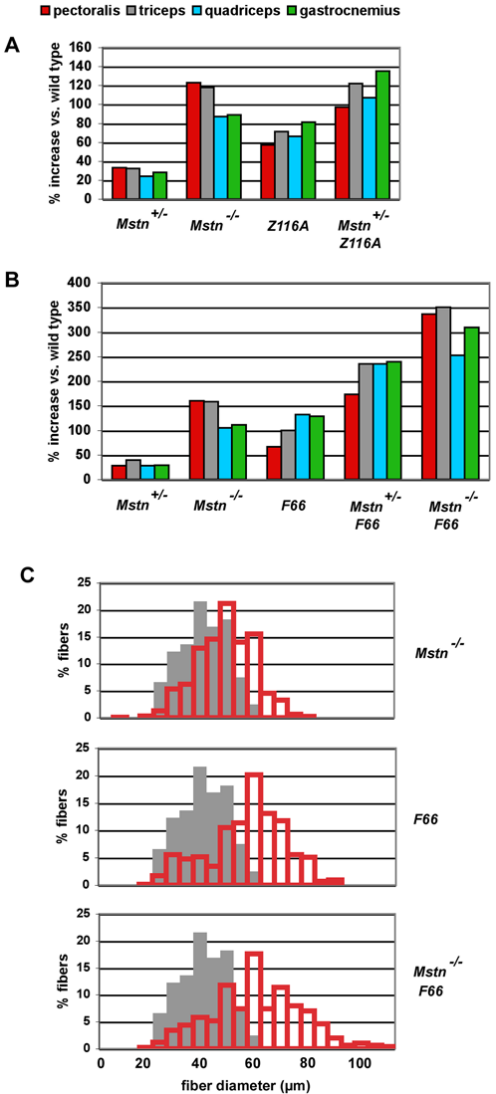
Muscle weight increases in (A) female *Mstn* mutant and *Z116A* transgenic mice and (B) male *Mstn* mutant and *F66* transgenic mice. Numbers represent percent increases relative to wild type mice and were calculated from the data shown in Table 1. (C) Distribution of fiber diameters. Gray bars represent muscles from wild type mice, and red bars represent muscles from *Mstn^−/−^*, *F66*, and *F66/Mstn^−/−^* mice.

To determine whether the FLRG transgene was causing increased muscle growth by blocking myostatin activity, I examined the effect of combining the FLRG transgene with a loss-of-function mutation in the myostatin gene. To date, using the Z116A line, I have not been able to generate mice that are both positive for the transgene and homozygous for the myostatin deletion mutation. However, I did obtain a number of female Z116A transgenic mice that were heterozygous for the myostatin mutation, and as shown in Table 1 and Figure 2a, these mice exhibited further increases in muscle weights compared to Z116A mice that were wild type for myostatin. Most importantly, in two of the muscles that were examined (quadriceps and gastrocnemius) the observed increases were also greater than those seen in *Mstn^−/−^* mice lacking the transgene. Based on this finding, it appears that myostatin cannot be the sole target for FLRG in the transgenic mice and, therefore, that additional ligands must be capable of suppressing muscle growth *in vivo*.

### Effect of follistatin in *Mstn* null mice

**Figure 3:**
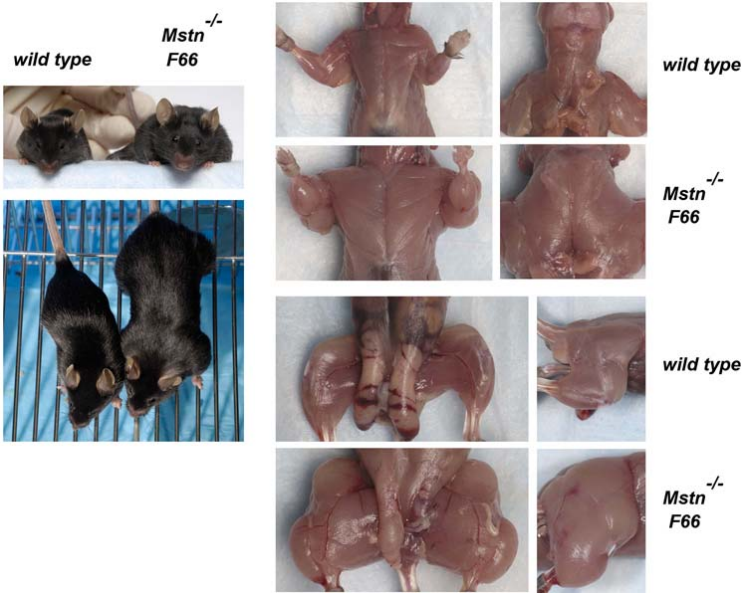
Comparison of wild type and *F66/Mstn^−/−^* mice.

Because I was unable to examine the effect of overexpressing FLRG in the complete absence of myostatin, it was difficult to ascertain the relative importance of these additional ligands compared to myostatin in regulating muscle mass. However, I carried out a similar set of experiments utilizing follistatin transgenic mice, which demonstrated that these additional ligands do play a major role in suppressing muscle growth. In previous studies, we had generated several transgenic founders expressing follistatin from a myosin light chain promoter/enhancer [19]. I was able to establish a transgenic line from one of these founders (*F66*), and I backcrossed this line extensively to C57 BL/6 mice for subsequent analysis. In this line, the transgene was most likely located on the Y chromosome, as the transgene was transmitted to all of the male offspring and none of the female offspring. *F66* transgenic mice were mated with *Mstn* mutant mice, and *F66*/*Mstn^+/−^* males were then mated with either *Mstn^+/−^* or *Mstn^−/−^* females. As shown in Table 1, the presence of one or two *Mstn* mutant alleles in combination with the *F66* transgene resulted in increasingly more muscle mass than seen in *F66* transgenic mice that were wild type for *Mstn*. Moreover, muscle weights in either *F66*/*Mstn^+/−^* or *F66*/*Mstn^−/−^* mice were dramatically higher than in *Mstn^−/−^* mice lacking the *F66* transgene. In the most extreme case, muscle weights in *F66*/*Mstn^−/−^* mice were increased by 250–350% from those seen in wild type mice (Figures 2b and 3). Hence, the presence of the *F66* transgene in a *Mstn^−/−^* background caused yet another doubling of muscle weights, resulting in mice with approximately quadruple the normal amount of muscle. These findings demonstrate that like FLRG, follistatin must be exerting its effect on muscle growth by targeting other ligands in addition to myostatin and that the effect of blocking these other ligands is comparable in magnitude to that resulting from loss of myostatin.

In previous studies, we showed that the increase in muscle mass in *Mstn^−/−^* mice results from a combination of increased fiber numbers and increased fiber sizes [2]. To determine whether the same is true for the additional muscle mass seen upon introduction of the *F66* transgene, I carried out morphometric analysis of the gastrocnemius/plantaris muscles. As shown in Table 2 and Figure 2c, total fiber number and mean fiber diameter were increased by about 48% and 19%, respectively, in *Mstn^−/−^* mice compared to wild type mice. As the cross-sectional area of the muscle would be expected to be roughly proportional to the square of the diameter, increased fiber diameter in *Mstn^−/−^* mice would correspond to an approximately 43% increase in fiber mass. Hence, muscle fiber hyperplasia and hypertrophy appear to contribute roughly equally to give the overall doubling of gastrocnemius/plantaris mass in *Mstn^−/−^* mice. In contrast, a similar analysis of *F66* transgenic mice revealed that although total fiber number was increased slightly (16%), the overall increase in gastrocnemius/plantaris mass resulted almost entirely from muscle fiber hypertrophy (93% increase in cross-sectional area). In mice in which the *F66* transgene was combined with the *Mstn* null mutation, the two phenotypes appeared to be additive; that is, the quadrupling of muscle mass in *F66*/*Mstn^−/−^* mice resulted from an approximately 73% increase in fiber number and 117% increase in fiber cross-sectional area. These results suggest that the additional muscle mass induced by follistatin in *Mstn* null mice results from inhibition of additional ligands that act predominantly to regulate muscle fiber growth.

**Table 2:** Morphometric analysis of gastrocnemius/plantaris muscles.

genotype	n	total fiber number	relative fiber number	mean fiber diameter (µm)	relative fiber diameter	relative cross-sectional area a
*Mstn* ***^+/+^***	4	8451±505	1.00	41.3±1.0	1.00	1.00
*Mstn* ***^−/−^***	4	12488±1251^b^	1.48	49.3±2.2 b	1.19	1.43
*F66, Mstn* ***^+/+^***	3	9838±84 b	1.16	57.4±1.1 c	1.39	1.93
*F66, Mstn* ***^−/−^***	2	14593±849 b	1.73	60.8±1.1 c	1.47	2.17

a calculated as relative fiber diameter squared,

b *p*<0.05 vs. *Mstn* ***^+/+^***,

c *p*<0.001 vs. *Mstn* ***^+/+^***

### Maternal effect of the *Mstn* null mutation

In the experiments with *F66* transgenic mice, a consistent finding was that muscle weights were higher in animals of the same genotype if they arose from crosses in which the mother had fewer functional *Mstn* alleles (Table 1). This maternal effect was observed to some extent in all of the muscles examined but was most pronounced in the quadriceps and gastrocnemius. For example, muscle weights of *F66*/*Mstn^+/+^* males obtained from crosses with *Mstn^+/−^* females were higher than those of *F66*/*Mstn^+/+^* males obtained from crosses with *Mstn^+/+^* females. Similarly, muscle weights of *F66*/*Mstn^+/−^* males obtained from crosses with *Mstn^−/−^* females were higher than those of *F66*/*Mstn^+/−^* males obtained from crosses with *Mstn^+/−^* females. The most dramatic effects were observed in *F66*/*Mstn^−/−^* mice obtained from crosses with *Mstn^−/−^* females, in which muscle weights were approximately quadrupled compared to wild type mice.

To determine whether this maternal effect was specific to the presence of the *F66* transgene, I carried out a variety of crosses of *Mstn* mutant mice lacking the transgene. As shown in Table 3, the maternal effect on muscle weights was observed in these crosses as well. In virtually every case, mice with identical genotypes exhibited higher muscle weights if the mother had fewer functional *Mstn* alleles. The most clear cut results were obtained in analyses of *Mstn^+/−^* offspring derived from crosses of *Mstn^+/+^* males with *Mstn^−/−^* females, which showed significantly higher muscle weights than *Mstn^+/−^* offspring derived from crosses of *Mstn^−/−^* males with *Mstn^+/+^* females. Hence, the maternal effect on muscle mass was not dependent on the presence of the F66 transgene.

**Table 3:** Maternal effect of *Mstn* null mutation.

line	*Mstn*	sex	father	mother	n	pectoralis	triceps	quadriceps	gastrocnemius
1	+/+	F	*Mstn^+/+^*	*Mstn^+/+^*	22	47.3±0.8	68.2±1.1	142.8±1.7	95.9±1.3
2	+/+	F	*Mstn^+/−^*	*Mstn^+/−^*	15	51.2±1.4 a	70.7±1.4	147.9±3.2	101.3±2.3 a
3	+/−	F	*Mstn^−/−^*	*Mstn^+/+^*	19	59.8±1.1	84.3±1.6	165.4±2.6	113.4±1.3
4	+/−	F	*Mstn^+/−^*	*Mstn^+/−^*	15	63.0±0.8 b	90.0±1.5 b	176.6±2.4 c	122.8±1.6 d
5	+/−	F	*Mstn^+/+^*	*Mstn^−/−^*	19	65.3±2.5 c	93.7±2.8 d	181.3±5.0 d	123.6±3.2 d
6	−/−	F	*Mstn^+/−^*	*Mstn^+/−^*	10	105.7±3.4	148.7±3.7	266.9±6.7	181.1±3.8
7	−/−	F	*Mstn^−/−^*	*Mstn^−/−^*	19	110.6±1.9	156.9±3.1	278.6±4.9	192.5±3.6 e
8	+/+	M	*Mstn^+/+^*	*Mstn^+/+^*	19	73.5±1.3	91.5±1.6	190.0±3.2	129.4±1.7
9	+/+	M	*Mstn^+/−^*	*Mstn^+/−^*	13	79.1±2.2 f	99.4±3.4 f	198.5±4.9	137.0±3.0 f
10	+/−	M	Mstn^−/−^	*Mstn^+/+^*	28	93.6±1.9	120.1±2.3	230.0±4.0	158.8±2.4
11	+/−	M	*Mstn^+/−^*	*Mstn^+/−^*	13	94.3±2.0	127.1±2.6	243.2±5.4	167.5±3.2 g
12	+/−	M	*Mstn^+/+^*	*Mstn^−/−^*	21	101.7±1.7 h	133.4±2.2 i	252.5±4.1 i	168.2±2.5 h
13	−/−	M	*Mstn^+/−^*	*Mstn^+/−^*	10	190.8±7.1	236.1±5.2	390.1±9.4	272.6±4.9
14	−/−	M	*Mstn^−/−^*	*Mstn^−/−^*	17	193.7±3.6	240.7±3.3	397.3±5.6	277.7±4.1

Numbers represent muscle weights (mg).

a *p*<0.05 vs. line 1,

b *p*<0.05 vs. line 3,

c *p*<0.01 vs. line 3,

d *p*<0.001 vs. line 3,

e *p*<0.05 vs. line 6,

f *p*<0.05 vs. line 8,

g *p*<0.05 vs. line 10,

h *p*<0.01 vs. line 10,

i *p*<0.001 vs. line 10

Conceivably, this maternal effect could result from transfer of myostatin or a downstream mediator either prenatally from the maternal to fetal circulations or postnatally from the mother to the offspring during nursing; in this respect, myostatin mRNA has been reported to be expressed in the mammary gland of lactating pigs [24]. To distinguish these two possibilities, I analyzed the effect of transferring neonates obtained from crosses with mothers of one *Mstn* genotype to foster mothers of a different *Mstn* genotype. In these experiments, all transfers were carried out using neonatal mice less than 24 hours old to mothers that had delivered their own litters also within the previous 24 hours. In order to control for effects of the transfer process *per se*, I also carried out transfers of neonates obtained from crosses with mothers of one *Mstn* genotype to foster mothers of the same *Mstn* genotype. As shown in Table 4, mice of a given genotype and parentage exhibited comparable muscle weights regardless of the genotype of the foster mothers. Hence, if there is a mediator of muscle mass that is transferred through the milk, I was not able to detect any resultant effects on muscle mass in these experiments. Taken together, these results suggest that the maternal effect on muscle mass results most likely from prenatal transfer of some mediator from mother to fetus, perhaps myostatin itself.

**Table 4:** Muscle weights (mg) following transfer of newborn mice to foster mothers.

*Mstn*	sex	parents	foster mother	n	pectoralis	triceps	quadriceps	gastrocnemius
+/+	F	*Mstn^+/+^*	*Mstn^+/+^*	8	47.8±1.4	66.0±1.1	143.3±4.2	97.0±2.5
+/+	F	*Mstn^+/+^*	*Mstn^−/−^*	19	47.9±0.9	66.8±0.7	143.2±1.9	97.1±1.2
+/+	M	*Mstn^+/+^*	*Mstn^+/+^*	10	73.7±2.5	92.9±3.0	191.0±7.4	129.3±4.3
+/+	M	*Mstn^+/+^*	*Mstn^−/−^*	15	74.5±1.6	91.5±1.8	192.8±3.6	129.6±1.9
−/−	F	*Mstn^−/−^*	*Mstn^+/+^*	13	115.9±2.9	156.6±3.3	280.8±7.2	191.8±4.2
−/−	F	*Mstn^−/−^*	*Mstn^−/−^*	11	110.5±1.9	150.5±2.1	281.0±4.8	194.5±2.9
−/−	M	*Mstn^−/−^*	*Mstn^+/+^*	15	189.5±5.0	215.9±5.3	376.2±7.2	260.3±4.8
−/−	M	*Mstn^−/−^*	*Mstn^−/−^*	14	193.5±3.7	218.4±4.5	385.4±7.3	267.2±5.0

## Discussion

Based on the data presented here, two important conclusions can be drawn. The first is that the *Mstn* loss-of-function mutation exerts a maternal effect such that muscle mass of the fetus is influenced by the number of functional *Mstn* alleles in the mother. Specifically, I show that offspring with identical *Mstn* genotypes have higher muscle weights if the mother has fewer functional *Mstn* alleles. This finding taken together with the results of cross fostering experiments suggest that muscle mass can be influenced by prenatal transfer of some mediator from mother to fetus; although myostatin itself is the most obvious candidate for this mediator, additional experiments will be required to prove this definitively.

We showed previously that myostatin circulates in the blood and that systemic effects can be achieved by implanting myostatin-expressing cells into a single site in the lower limb [21]; however, there have been no experiments that have demonstrated conclusively that myostatin normally acts systemically. The demonstration that maternal effects can be seen in offspring of mice lacking myostatin is consistent with the possibility that the circulating myostatin protein can enter the active pool. If myostatin does act systemically, the implication would be that local control of muscle growth can be influenced at least in part by myostatin being produced elsewhere in the body and that myostatin functions precisely as a chalone, as originally hypothesized by Bullough [25], [26] for the control for tissue growth in general. This is a critical issue, as it relates to the fundamental reason that the control of muscle growth may have evolved into this rather complex regulatory system. In this respect, I speculated previously that perhaps the myostatin regulatory system may serve two distinct functions, one to regulate local growth of muscle in response to specific physiological stimuli, such as injury, and a second to regulate the overall metabolic balance between fat and muscle in response to general physiological stimuli, such as nutritional status [1]. According to this model, local control of muscle growth would be achieved by regulating the extent to which the latent form of myostatin is activated at the target site, whereas global control of the metabolic homeostatic balance between muscle and other tissues would be achieved by regulating the size of the circulating pool of myostatin.

The second important finding presented here is the demonstration that other ligands work with myostatin to control muscle growth. I have presented data showing that FLRG, like follistatin, can promote muscle growth when expressed as a transgene in skeletal muscle and that both of these molecules appear to act by blocking not only myostatin but also other ligands with similar activity to myostatin. By combining the follistatin transgene with a myostatin null mutation, I have been able to generate mice with quadrupled muscle mass, which represents yet another doubling of muscle mass compared to mice only lacking myostatin. These studies demonstrate that muscle mass in mice is controlled by multiple members of the transforming growth factor-ß superfamily acting in concert. We reached a similar conclusion in an earlier study in which we demonstrated that administration of a soluble form of the ACVR2B receptor to wild type could cause more extensive muscle growth than what had been observed previously using myostatin-specific inhibitors and that this soluble receptor could also increase muscle growth even in mice completely lacking myostatin [7]. The studies presented here demonstrate that the capacity for promoting muscle growth by targeting this general signaling pathway is far greater than previously appreciated.

Because myostatin normally acts to limit muscle growth, there has been considerable interest in targeting this pathway to attempt to enhance muscle growth in human patients with muscle wasting and muscle degenerative diseases. Most efforts in this regard have focused on agents capable of binding specifically to myostatin and inhibiting its activity. The finding that myostatin is not the sole regulator of muscle mass in mice raises the question as to whether targeting myostatin alone will be the most effective strategy for manipulating this signaling pathway in humans. In this respect, it is known that the circulating levels of myostatin protein in humans are considerably lower than in mice [14], [21], raising the possibility that the balance of the relative roles played by myostatin and by these other regulators may have shifted further away from myostatin in humans compared to mice. For these reasons, it will be essential to determine the identity of the other ligand or ligands that cooperate with myostatin, as only then will we be able to develop the best possible strategies for manipulating this pathway for the treatment of human diseases in which promoting muscle growth may be beneficial.

## Materials and Methods

Pronuclear injections of DNA and embryo transfers were carried out by the Johns Hopkins Transgenic Core Facility. *Mstn* mutant and *F66* and FLRG transgenic mice were backcrossed at least 6 times onto a C57 BL/6 background prior to analysis. All analysis was carried out on 10 week old mice. For measurement of muscle weights, individual muscles from both sides of the animal were dissected, and the average weight was used for each muscle. For morphometric analysis, the gastrocnemius and plantaris muscles were sectioned serially to their widest point using a cryostat, and fiber diameters were measured (as the shortest distance across the fiber passing through the midpoint) from hematoxylin and eosin stained sections. Measurements were carried out on 250 fibers per animal, and all data for a given genotype were pooled.
